# Comparison of the Differences between Two-Photon Excitation, Upconversion, and Conventional Photodynamic Therapy on Cancers in In Vitro and In Vivo Studies

**DOI:** 10.3390/ph17060663

**Published:** 2024-05-21

**Authors:** Chuanshan Xu, Siu Kan Law, Albert Wing Nang Leung

**Affiliations:** 1 Guangzhou Municipal and Guangdong Provincial Key Laboratory of Molecular Target & Clinical Pharmacology, The NMPA and State Key Laboratory of Respiratory Disease, School of Pharmaceutical Sciences, Guangzhou Medical University, Guangzhou 511436, China; 2 Department of Food and Health Sciences, The Technological and Higher Education Institute of Hong Kong, Tsing Yi, New Territories, Hong Kong; siukanlaw@hotmail.com; 3 School of Graduate Studies, Lingnan University, Tuen Mun, Hong Kong; albertleung@ln.edu.hk

**Keywords:** two-photon excitation, upconversion, nanoparticle, photodynamic therapy, cancer

## Abstract

Photodynamic therapy (PDT) is a minimally invasive treatment for several diseases. It combines light energy with a photosensitizer (PS) to destroy the targeted cells or tissues. A PS itself is a non-toxic substance, but it becomes toxic to the target cells through the activation of light at a specific wavelength. There are some limitations of PDT, although it has been used in clinical studies for a long time. Two-photon excitation (TPE) and upconversion (UC) for PDT have been recently developed. A TPE nanoparticle-based PS combines the advantages of TPE and nanotechnology that has emerged as an attractive therapeutic agent for near-infrared red (NIR) light-excited PDT, whilst UC is also used for the NIR light-triggered drug release, activation of ‘caged’ imaging, or therapeutic molecules during PDT process for the diagnosis, imaging, and treatment of cancers. Methods: Nine electronic databases were searched, including WanFang Data, PubMed, Science Direct, Scopus, Web of Science, Springer Link, SciFinder, and China National Knowledge Infrastructure (CNKI), without any language constraints. TPE and UCNP were evaluated to determine if they had different effects from PDT on cancers. All eligible studies were analyzed and summarized in this review. Results: TPE-PDT and UCNP-PDT have a high cell or tissue penetration ability through the excitation of NIR light to activate PS molecules. This is much better than the conventional PDT induced by visible or ultraviolet (UV) light. These studies showed a greater PDT efficacy, which was determined by enhanced generation of reactive oxygen species (ROS) and reduced cell viability, as well as inhibited abnormal cell growth for the treatment of cancers. Conclusions: Conventional PDT involves Type I and Type II reactions for the generation of ROS in the treatment of cancer cells, but there are some limitations. Recently, TPE-PDT and UCNP-PDT have been developed to overcome these problems with the help of nanotechnology in in vitro and in vivo studies.

## 1. Introduction

Photodynamic therapy (PDT) is a less invasive treatment for cancer. This is usually utilized as a supplementary therapy applied before or after chemotherapy, ionizing radiation, or surgery. PDT has the selectivity to kill the tumor cells or tissues and can be repeated many times in the same area, which is different from radiotherapy and surgery. It is an effective and reliable approach [1]. However, PDT has some disadvantages in treating large, deeply hidden tumors, because it is difficult to efficiently excite the photosensitizer (PS) for locations more than 1 cm deep [2,3]. Thus, two-photon excitation (TPE) and upconversion nanoparticles (UCNPs) in PDT using near-infrared light as the activating source have been recently developed.

In TPE-PDT, the photosensitizer (PS) can simultaneously absorb two photons, and be promoted from the ground to the excited electronic state under near-infrared (NIR) light excitation. The excited molecules then react with oxygen to produce reactive oxygen species (ROS) including singlet oxygen (^1^O_2_), superoxide ions (O_2_^−^), free radicals (O_2_^•−^, •OH), etc. This avoids the absorption and scattering in the epidermal tissue to treat deeper tissues, such as tumors [4].

In UCNP-PDT, PS is activated by near-infrared (NIR) light, which is coated on the nanoparticles, resulting in the generation of ROS and the direct destruction of the tumor [5]. TPE and UCNP using nanocarriers combined with PS enhance its accumulation in the tumor site, owing to preferential extravasation of nanoparticles into the tumor vasculature by increasing the permeability and retention effect [6].

Conventional PDT continues to evolve as a promising cancer treatment strategy, with ongoing research planning from fundamental investigations to clinical trials, exploring various photosensitizers and treatment combinations [7]. However, there have been some limitations in the clinical trials for a long time. The present article aims to compare the differences between TP, UC, and conventional PDT against cancers in clinical studies. This review is divided into three parts, consisting of a background principle, the research progress of TPE-PDT and UCNP-PDT to combat cancers as well as the comparison between these therapies.

### 1.1. Basic Principles of Conventional PDT

**Figure 1:**
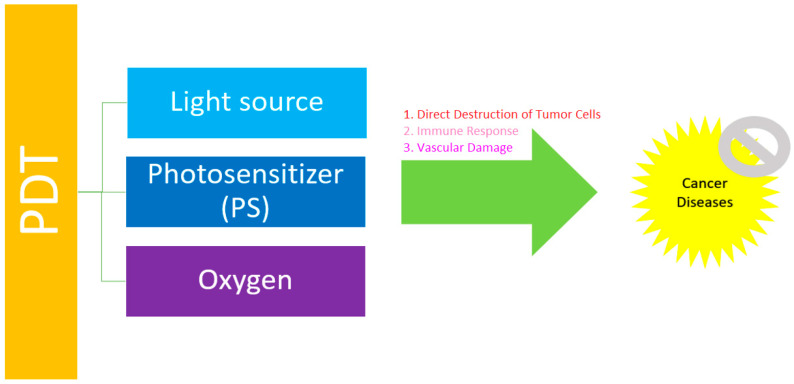
The general principle of PDT in the treatment of cancer diseases.

PDT combines three elements, including the use of a PS, light, and molecular oxygen (O_2_) [8]. Generally, a PS absorbs light producing the excited species and reacts with O_2_ to generate highly ROS. These ROS cause an imbalance of the redox state in tumor cells leading to cell death [9] (Figure 1). Actually, PS is irradiated with visible light of a suitable wavelength to absorb the energy from a ground state S_0_ to an excited state S_1_ (singlet). It involves “Type II Redox Reaction” and “Type I Redox Reaction”.

**Figure 2:**
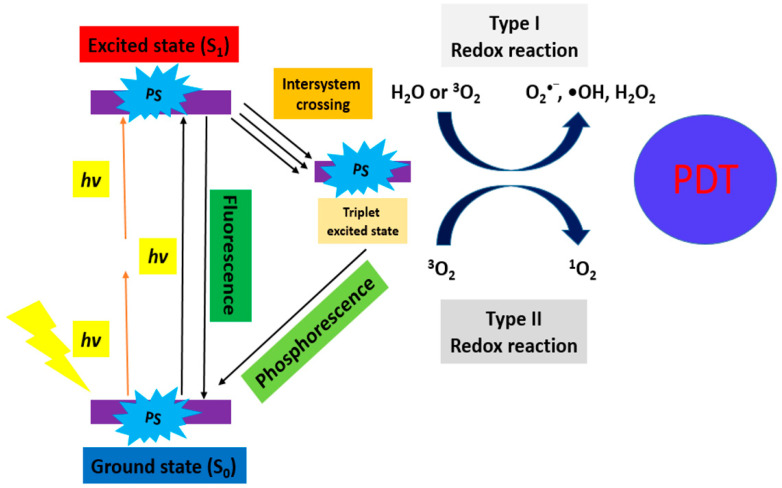
Schematic diagram of the excitation processes for conventional PDT (one-photon) and TPE-PDT (two-photon, orange in color) [11].

**Figure 3:**
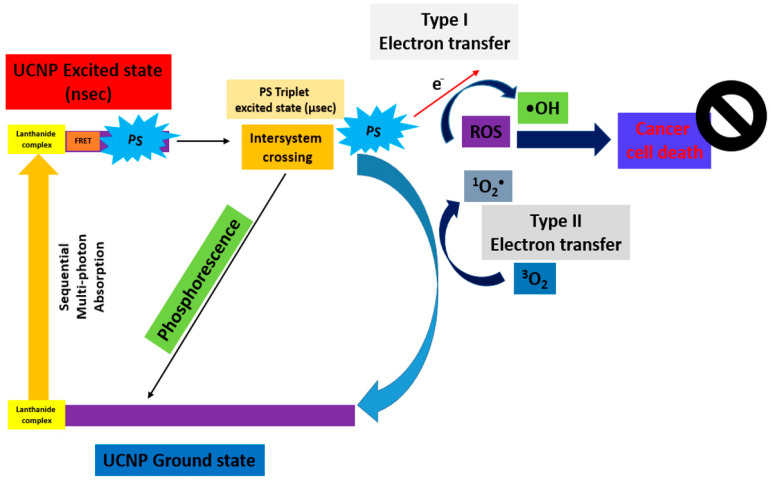
Schematic diagram of the excitation processes for UCNP-PDT [12].

“Type II Redox Reaction” consists of the energy transfer, and uses the ^3^O_2_ in its ground state as the acceptor of energy forming ^1^O_2_. “Type I Redox Reaction” involves the formation of superoxide ions (O_2_**^−^**), free radicals (O_2_^•−^, •OH), and H_2_O_2_ from the water and ^3^O_2_. These mechanisms simultaneously occur, and the formation of ROS depends on the PS type and concentration, or oxygen [10]. This principle is further demonstrated in Figure 2 (TPE-PDT) [11] and Figure 3 (UCNP-PDT) [12].

### 1.2. PDT on Cancers

Cancer is the first or second leading cause of premature death worldwide. According to the most updated information from the Hong Kong cancer registry, nearly 35,000 diagnosed cancer cases in 2020 were recorded. There were around 15,000 registered cancer deaths, and lung cancer was the most common cancer in both genders, followed by colorectal, liver, pancreatic, and breast cancers [13]. Up until the present, an increase in adult population size has been the major determinant for incremental cancer deaths in different countries [14].

PDT is possible to fit into cancer care, which prolongs the survival of the inoperable cancer patient and significantly improves their quality of life [15]. In patients with early-stage lung cancer, PDT can eliminate cancerous cells before tumors grow or spread. Patients with large lung tumors are required to use PDT for shrinking the tumor before surgery to relieve the pressure on nearby organs, which reduces the risk of shortness of breath in the patient with lung cancer [16]. In addition, PDT is also suitable for the treatment of other cancers, such as colorectal, liver, pancreatic, and breast cancers, as shown through in vitro or in vivo studies (Table 1). There are three types of mechanisms of PDT against cancer: direct destruction of tumor cells, immune response, and vascular damage.

#### 1.2.1. Direct Destruction of Tumor Cells

There are several routes for PS uptake in cancer cells, such as phagocytosis or endocytosis, low-density lipoprotein receptor binding, lipid binding, uptake via tyrosine kinase or epidermal growth factor receptor, diffusion, and bio-distribution [17]. When higher light energy is applied, it causes damage to cellular, or sub-cellular membranes. The cancer cells are ablated via necrosis [18]. Conversely, apoptotic death may be initiated by PDT when a low intensity light is employed. Cancer cells cease functioning with no bystander effect or immune response during the apoptotic pathway, which directly eliminates cancer cells via the light activation of the PS [19].

#### 1.2.2. Immune Response

PDT induces necrosis of tumors and their vasculature, which also initiates an immune reaction cascade [20]. It releases inflammatory mediators, such as cytokines, growth factors, and proteins. The action of PDT stimulates white blood cells, including neutrophils, and activates macrophages to kill tumor cells through CD4 helper T lymphocytes and activate CD8 cytotoxic T lymphocytes [21,22].

#### 1.2.3. Vascular Damage

The PS can accumulate in cancer cells in the vascular system [23]. When PS is activated by the appropriate light, photodynamic action is induced. This affects the permeability of vascular walls, and platelets become aggregated and result in blood clotting [24]. Tumor regions lack blood supply and cause cell death of cancer cells [25]. This also releases toxic substances such as thromboxanes and toxic cytokines to activate the immune system [26].

**Table 1:** PDT for the treatment of different cancers either in in vitro or in vivo studies.

**In Vitro**					
	**Study**	**Photosensitizer (PS)**	**Usage of Light and** **Energy (J)**	**Consequence**	**Reference**
1	Genetic aberrations associated with photodynamic therapy in colorectal cancer cells	Zinc (Zn) metal-based phthalocyanine (ZnPcSmix)	Laser at 680 nm with 5 J/cm^2^	Lysosomal initiation of apoptotic cell death in response to PDT, which delayed mitochondrial cytochrome C leakage as induced by the proteolytic enzyme cathepsin D as well as decreased pH in the lysosomes.	[27]
2	Anti-cancer effects of oncolytic viral therapy combined with photodynamic therapy in human pancreatic cancer cell lines	Protoporphyrin IX (PpIX)	A red light-emitting diode at 653 nm with 0.54 J/cm^2^	Reovirus with PpIX-mediated photodynamic therapy resulted in a significantly increased cytotoxic effect, and the photodynamic therapy with 100% cell death was observed in pancreatic cell lines.	[28]
3	Methylene blue photodynamic therapy induces selective and massive cell death in human breast cancer cells	Methylene blue	A light-emitting diode (LED) array at 640 nm with 4.5 J/cm^2^	Methylene blue-PDT increased the eradication rate of microscopic residual disease, thus minimizing the chance of both local and metastatic recurrence.	[29]
**In Vivo**					
1	Photodynamic therapy using methylene blue in lung cancer animal models	Methylene blue	Intra-tumoral injection and irradiation to laser at 630 nm with 200 J/cm^2^	Methylene blue was inexpensive and efficient as a PDT agent for lung cancer treatment but the safety and efficacy required further study.	[30]
2	Photodynamic therapy (PDT) for lung cancers	Photofrin; mono-l-aspartyl chlorine e6 (NPe6)	Laser at 640 nm or 664 nm with 100 to 200 J/cm^2^ for 40 mg/m^2^ intravenous administration.	PDT successfully either reduced the extent of resection or increased operability.	[31]
3	Clinical trial of photodynamic therapy for peripheral-type lung cancers using a new laser device in a pilot study	Talaporfin sodium	Laser at 664 nm with 120 mW/cm^2^	PDT was found to be a feasible and non-invasive treatment modality for early peripheral-type lung cancer.	[32]
4	Photodynamic therapy for colorectal cancer: A systematic review of clinical research	Hematoporphyin derivative or Photofrin	A laser at 630 nm with 50 to 100 J/cm^2^	PDT for the management of colorectal cancer was not well studied, it required establishing and defining the role of PDT in the management of colorectal cancer.	[33]
5	Photodynamic therapy of colorectal cancer using a new light source	Photofrin II^®^	A Versa-Light^®^ at 630 nm with 50 to 500 J/cm^2^	Versa-Light^®^ was a good light source for PDT, and effective in both in vitro and animal studies.	[34]
6	Application of photodynamic therapy for liver malignancies	Talaporfin sodium	A laser at 664 nm with 40 mg/m^2^	PDT was considered a promising treatment modality for all liver cancers, but several challenges still impede the application of PDT in liver malignancies.	[35]
7	Photodynamic therapy for cancer of the pancreas	Meso-tetrahydroxyphenyl chlorin (mTHPC)	A diode delivering red light at 652 nm with 100 mW/cm^2^	PDT was leading the necrosis in pancreatic cancer cells although care was required for tumors invading the duodenal wall or involving the gastroduodenal artery.	[36]
8	Phase I/II study of verteporfin photodynamic therapy in locally advanced pancreatic cancer	Meso-tetrahydroxyphenyl chlorin (mTHPC)	A laser at 690 nm with 150 mW/cm^2^	Verteporfin PDT-induced tumor necrosis in locally advanced pancreatic cancer is feasible and safe.	[37]
9	Photodynamic therapy in primary breast cancer	Verteporfin	A laser at 690 nm with 150 mW/cm^2^	PDT was a safe, and minimally invasive treatment for primary breast cancer that was reasonably predictable with minimal side effects on normal tissue compared to other local therapies.	[38]
**In Vitro and In Vivo**					
1	Selective accumulation of ALA-induced PpIX and photodynamic effect in chemically induced hepatocellular carcinoma	5-aminolevulinic acid (ALA)	Intravenous administration 3 h before laser irradiation at 630 nm with 160 mW/cm^2^	The interstitial irradiation of ALA-PDT was an effective treatment for hepatocellular carcinoma.	[39]
2	Photodynamic treatment with purpurin 18 effectively inhibits triple-negative breast cancer by inducing cell apoptosis	Purpurin 18	A laser at 660 nm with 600 J/cm^2^	Intra-tumoral pu-18-PDT treatment had high photodynamic efficacy and low toxicity, which inhibited the growth of triple-negative breast cancer by inducing the apoptosis of cancer cells.	[40]

### 1.3. Limitations of Conventional PDT

Some limitations of conventional PDT exist in vitro, in vivo, and in clinical studies. The therapy is hindered by the properties of PS, including water solubility, limited light-penetration depth, and poor tumor targeting efficiency [41,42].

For the clinical PDT studies, the photodynamic effect takes place selectively at a diseased site and is highly dependent on the oxygen level in tissues. Treatment efficacy relies on accurate light delivery to the tumor. In general, deep tumors are difficult to treat because of the poor penetration of visible light in the biological tissues [43,44]. Furthermore, the procedures of PDT are multiple and complex. Patients may experience photosensitivity issues, e.g., skin allergy, within a few weeks after therapy [45,46].

The light penetration depth in human tissue and the ability to illuminate the entire tumor homogeneously are important issues in PDT [47]. Conventional PDT cannot treat superficial tumors, which are deeper than 1 cm. Interstitial PDT or a combination with debulking surgery is a possible way to reach deeper tumors [48]. The selectivity of PS is another serious problem of PDT because its accumulation and localization within the tumor affect the generation of ROS upon light irradiation [49].

## 2. Two-Photon Excitation for PDT (TPE-PDT)

TPE-PDT uses near-infrared light as an excitation source and the wavelength is usually greater than 700 nm. It is an essential approach for modern deep-tissue phototherapy compared to traditional PDT. Two-photon offers the advantage of enhanced spatial precision, which enables targeted treatment without harming the surrounding healthy tissue [50].

The general mechanism of TPE-PDT is a nonlinear absorption, consisting of two relatively low-energy photons. After absorbing low-energy NIR light, TPE can emit high-energy photons. This high-energy light can excite PS to produce cytotoxic ROS to kill cancer cells [51].

In early 1997, Bhawalkar et al. demonstrated the use of infrared excitation in conjunction with an efficient two-photon absorbing dye and a photosensitizer in PDT. They discovered the two-photon absorbing dye, including 4-[N-(2-hydroxyethyl)-N-(methyl) amino phenyl]-4′-(6-hydroxyhexyl sulfonyl)stilbene (APSS), exhibited a strong two-photon absorption at 800 nm, and upconverted fluorescence at 520 nm in the solution to generate ^1^O_2_ under infrared excitation at 800 nm. The characteristic of two-photon excitation is that two-photon absorbing dye contains a “chromophore”, which acts as a “photon harvester”. The function of the chromophore is to extend an original wavelength to the near-infrared spectral region and transfer the energy to the PS for generating ^1^O_2_ [52]. There are some examples of using TPE-PDT for the treatment of different cancers (Table 2).

## 3. UpConversion Nanoparticles (UCNPs) of PDT

UCNPs can emit high-energy light under near-infrared (NIR) light excitation, which activates PS molecules to produce ROS and kill cancer cells [68]. Compared to traditional PDT, UCNPs often have higher photochemical stability, and UCNP-based PDT exhibits improved tissue penetration depth. They are usually lanthanide-doped nanocrystals, which emit high-energy photons under excitation by the NIR light. The energy of a photon depends on the synthesis of UCNPs related to the size and emission spectra [69].

There are three major components in UCNPs, including the matrix, sensitizer, and activator. The matrix should have modest phonon energy to avoid non-radiative relaxations, free NIR photon migration in the lattice, as well as chemical and thermal stabilities for crystal structure. Lanthanide dopants have narrow bandwidth emission, excellent chemical stability, and photostability, and low toxicity to tissues [70]. Europium (Eu) and Ytterbium (Yb) are widely utilized as a sensitizer because of the high energy level in f–f transitions of lanthanide [71].

UCNP-based PDT has also been demonstrated in in vitro and in vivo studies. For example, KillerOrange-Mito (mtKO) is gene-modified by triphenylphosphine (TPP). MitKO as PS with good targeting ability to mitochondria in cancer cells has been used in imaging-guided drug delivery and photothermal therapy [72].

In most cases of UCNPs, high and continuous light excitation are often required to obtain the necessary upconversion emission intensity. However, the high power dosage of laser irradiation could produce substantial heat, which in turn causes undesirable cell or tissue damage. Thus, it is required to decrease the power density of the laser and its accompanying heating effects while guaranteeing the desired emission intensity [73]. There are some examples of utilizing UCNP-PDT for the treatment of different cancers (Table 3).

## 4. Discussion

Based on Table 1, Table 2 and Table 3 data, we summarize that the usage of the wavelength in conventional photodynamic therapy is shorter than TPE-PDT and UCNP-PDT. Meanwhile, the usage of energy in conventional photodynamic therapy is greater than TPE-PDT and UCNP-PDT. Conventional photodynamic therapy uses greater energy, which may cause painful and unnecessary cell or tissue damage to patients, thus, TPE-PDT and UCNP-PDT are comparatively safe because of the lesser energy required.

### 4.1. Differences between TP and UC

Two-photon (TP) absorption involves the simultaneous absorption of two infrared photons of an atom or a molecule from the ground state to excite an electron, via a virtual energy level to a higher energy state; subsequent electron relaxation is accompanied by the emission of a photon with the shorter wavelength [85]. Meanwhile, two-photon excitation (TPE) occurs at the excited state, which is produced by two-photon absorption (TPA).

Upconversion (UC) is triggered by simultaneous and sequential two-photon absorption (TPA), which leads to the emission of light at a shorter wavelength than the excitation [86]. This is an anti-stokes emission that is the energy difference between the emitted and absorbed photon. Typically, the emitted photon has more energy than the absorbed photon [87]. The common ion used in photon upconversion is an f-block element, such as Ln^3+^, since it has large stokes’ shift and narrow emission bands [88].

### 4.2. Advantage of TPE-PDT and UCNP-PDT

Generally, compared to conventional PDT, TPE-PDT and UCNP-PDT have the potential to treat deeper tumors and/or improve tumor targeting with minimum damage to the surrounding healthy tissue [89].

TPE-PDT is a treatment technology with deep penetration and less damage, providing a broad prospect for cancer treatment. The development of TPE-PDT suffers from the low two-photon absorption (TPA) intensity and short triplet state lifetime of PS used in TPE-PDT [90]. Transition metal complexes such as platinum (Pt), palladium (Pd), and lanthanide (Ln) usually act as the PS in TPE-PDT because they have strong spin-orbit coupling between singlet and triplet states, making their inter-system crossing rate much higher than conventional organic molecules. This also produces a high quantum yield of ROS [91].

Wang et al. developed a naphthalimide-modified cyclometalated platinum(II) complex (PtPAN), which generated ROS efficiently under both normoxia and hypoxia. PtPAN showed low cytotoxicity in the dark and did not interact with DNA, but under the irradiation of near-infrared light at the wavelength of 825 nm the complex generated the ROS to specifically kill tumor cells [92]. Mariz et al. reported dipolar, or quadrupolar quinolizinium and benzimidazolium cations as precision photosensitizers with mitochondria targeting ability, which localized light-induced mitochondria damage in live animal cells under two-photon excitation in the NIR [93]. Chennoufi et al. also reported triphenylamine targeted cytosolic organelles of living cells, mainly mitochondria, triggering a fast apoptosis upon two-photon excitation [94].

Higher selectivity is the great advantage of TPE over one-photon absorption PDT. The femtosecond pulsed laser is more suitable for TPE-PDT than pico- and nanosecond pulses because the femtosecond pulsed laser has a high peak power pulse energy with less collateral damage [95]. However, most photosensitizers were used in the past with cross-section values less than 50 GM (One GM is 10^−50^ cm^4^·s·photon^−1^) and resulted in a low PDT efficiency under two-photon irradiation conditions [96].

UCNP can serve as an imaging probe and a drug carrier simultaneously. It exhibits anti-stokes luminescent that convert the NIR light into visible light. Meanwhile, UCNPs have high chemical stability, which can serve as fluorescence probes in a variety of complex organisms, facilitating the bioassay process, as well as cancer treatment [97]. UCNPs are often lanthanide-doped crystals that can absorb and convert low-energy NIR photons at the wavelength of 980 nm into high-energy UV-visible light [69].

Guan et al. designed a multifunctional NIR-triggered theranostic agent based on UCNP-Polyoxyethylene bis(amine)–trismethylpyridylporphyrin–fullerene nanocomposite (UCNP–PEG–FA/PC_70_) with β-NaGdF_4_, and Yb or Tm nanoparticles for imaging-guided photodynamic therapy for cancer. The light transducers converted NIR light into ultraviolet-visible light to activate PC_70_ for generating ^1^O_2,_ while UCNP–PEG–FA/PC_70_ was irradiated the wavelength of 980 nm laser with 0.8 W/cm^2^. Its enhanced permeability and retention effect improved the accumulation of multifunctional nanoparticles in tumors [98].

Moreover, Song et al. developed a feasible UCNP-based optogenetic nanosystem for NIR-triggered PDT and gene therapy for malignant tumors. The gene KillerOrange-Mito (mtKO) modified triphenylphosphine (TPP) as PS with good targeting ability to mitochondria in cancer cells. Then, UCNPs as the excitation source and gene carriers were developed. The siRNA specific to mRNA of B-cell leukemia (Bcl-2) was covalently modified onto the surface of UCNPs with the sandwich-like structure of NaYF_4_@NaYF_4_:Yb/Tm@NaYF_4_ via the ROSs-sensitive bond. The UCNPs with the genetically encoded PS into the mitochondria were then activated in situ via the luminescence resonance energy transfer (LRET) process under the excitation of NIR laser at the wavelength of 980 nm with 29 W/cm^2^, which induced a high cell apoptosis ratio of 60% and an obvious inhibition of the tumor volume [99]. Recently, Yang et al. demonstrated a facile strategy to switch the near-infrared emission at 800 nm from rationally designed UCNPs by modulating the irradiation laser into pulse output for photodynamic therapeutics [100].

The great advantage of UCNP-PDT is that UCNPs are employed as light transducers to convert NIR light into UV-to-visible light to effectively activate PS to produce ROS for killing tumors [101]. Meanwhile, UCNP is encapsulated in polymers, liposomes, and biomimics that improve the drug’s solubility and bioavailability to increase the effectiveness of PDT.

The efficacy of TPE-PDT or UCNP-PDT depends on ROS generation. TPE is attributed to its distinct photon upconverting feature, while UCNP is related to nanoparticles. The average quantum yield of ^1^O_2_ in TPE-PDT is 69.3 ± 10.0 GM [102], and the average quantum yield of luminescence in UCNP-PDT is 3.2% in red emission [101]. Both TPE-PDT and UCNP-PDT enhanced the ROS generation of PS under light irradiation at the longer wavelength and increased the effectiveness of PDT on deeper tumors [103].

A growing amount of evidence has shown that the performance of UCNP-PDT is a little bit better than the TPE-PDT. This is because UCNP is a nanoparticle doped with rare-earth lanthanide elements, such as Eu, Yb, Er, etc. These can be excited by more than 900 nm NIR light and emit 660 nm light, much longer than the TPE-PDT. UCNP also transforms externally applied NIR light into localized UV-Vis light to activate the PS during the PDT process.

### 4.3. Research Progress of TPE-PDT and UCNP-PDT

Conventional PDT may induce early and late-onset side effects including erythema, pain, burns, edema, itching, desquamation, and pustular formation because of exposure to the light source in the hours/days immediately after the therapy [104]. These are important problems in the clinical studies. Moseley et al. described the improvement of dosimetry to ensure an optimal light dose be delivered to tumors [105]. TPE-PDT is a safe and effective antitumor therapy that can be combined with surgery or immunotherapy. Wang et al. reported that aggregate-induced emission luminogens (TPE-IQ-2O) of PDT can not only reduce tumor recurrence in surgical treatment but also effectively improve the response to immune checkpoint inhibitors (ICIs) in immunotherapy without obvious toxicity. It increased CD8+ tumor-infiltrating lymphocyte accumulation to enhance the antitumor immunity [106]. Dobos et al. developed an in vitro screening platform for TPE-PS using a 3D osteosarcoma cell culture. The platform was tested using three different two-photon (2P) active compounds including a 2P sensitizer of water-soluble benzylidene cycloketone-based two-photon photoinitiator (P2CK), a fluorescent dye Eosin Y, and a porphyrin derivative (TPP), which showed no signs of damage to the surrounding healthy cells after TPE-PDT [107]. Wang et al. reported that TPE-PDT can occur in a low femtosecond laser power density. It conjugated carbon nanodots (cdots) with 5,10,15,20-tetrakis (1-methyl 4-pyridinio) porphyrins (TMPyP) and utilized FRET characteristics from cdots to TMPyP to produce ^1^O_2_ under irradiation of an NIR femtosecond laser [106]. Xu et al. developed a multifunctional NIR-II phototheranostic platform using a novel AIE-based dye (ZSY-TPE) for single laser-activated imaging-guided combined photothermal and photodynamic therapies of tumors and pathogens without any side effects [108]. Recently, Gong et al. reported a novel non-synthetic method taken to harness the synergistic therapeutic potential stemming from intermolecular interactions by employing nanoparticle encapsulation and a core–shell structure. The nanoparticle encapsulating exhibited low cytotoxicity, efficient antitumor properties, and excellent visualization [109].

UCNP-PDT converted NIR radiation into shorter wavelength visible and ultraviolet (UV) radiation; this is much better than conventional UV-activated tumor therapy that cannot be damaging to healthy surrounding tissue [110]. Punjabi et al. developed biocompatible upconverting nanoparticles (UCNPs) with largely amplified red emissions. This UCNP-PDT system with NIR irradiation outperforms clinically used red light irradiation in a deep tumor setting in vivo, and effectively accesses deep-set tumors [111]. Zhang et al. developed a novel nanoconjugate (UCNP-Ce6/AIEgen) for dual-pathway reinforced PDT, in which the UCNPs were co-modified with chlorin e6 (Ce6) and luminogen with aggregation-induced emission (AIEgen). This converted upconversion luminescence of UCNPs to the light that can activate Ce6 through Förster resonance energy transfer to generate more ROS, thus promoting tumor cell apoptosis [112]. Liu et al. indicated the UCNP as a core, and light-sensitive conjugated polymer and apo-transferrin-titanocene (Tf@Tc) as shells. Under NIR irradiation, apparent energy transfer occurs from the core to the polymer and Tc components in the shell, producing reactive oxygen species and free radicals for cancer cell killing [113]. Wang et al. designed a brain-targeting NIR theranostic system with a dual-site FRET route and superior spectral matching to maximize energy utilization for synergistic photodynamic and photothermal therapy of glioma [114].

### 4.4. Toxicity of TPE-PDT and UCNP-PDT

The toxicity of nanoparticles depends on their surface, particle size, particle morphology, and dissolution of ions [115]. Nanotoxicology of TPE and UCNPs is related to nanoparticle distribution, excretion, metabolism, pharmacokinetics, and pharmacodynamics in animal models. The toxicity of TPE is caused by the absorbing nanoparticles, such as gold nanorods, CdSe quantum dots, or carbon dots, because they are non-biodegradable [116]. This is closely associated with their surface chemistry and biocompatible surface coatings that significantly reduce their cytotoxicity [94]. It is much better to boost the therapeutic efficacy at a lower dose while promoting a biocompatible carrier material and decrease toxic side effects. PEGylation can improve the stability and minimize the toxicity of the UCNP-PS complex.

Meanwhile, Nahorniak et al. reported the cytotoxicity determination in rat mesenchymal stem cells by using the MTT assay, and showed that neutralization of the large positive surface charge of neat UCNPs with PEG-alendronate and the bound Rose Bengal sensitizer significantly reduced the concentration-dependent cytotoxicity [117]. Therefore, the potential risks in the clinical application of TPE and UCNPs are their systemic toxicity, the complexity of clearance, and long-term effects on the human body.

## 5. Conclusions

Conventional PDT involves “Type II and Type I redox reactions” for the generation of ROS in the treatment of cancer cells. Three types of mechanisms for PDT against cancer include the direct destruction of tumor cells, immune response, and vascular damage. However, there are some limitations of conventional PDT. The properties of PS are hindered by water solubility, limited light penetration depth, and poor tumor targeting efficiency. TPE-PDT and UCNP-PDT overcome these problems with the help of nanotechnology in either in vitro or in vivo studies. These modified the PS to have a longer absorption wavelength for the generation of ROS and increased the effectiveness of PDT on deeper tumors. TPE increases or improves the selectivity and specificity of a PS to tumor cells by targeting the recognition groups that can be introduced onto the PS. It also increases the therapeutic efficiency for solid tumor treatment. Thus, the development of TPE-PDT and UCNP-PDT draws the scientist’s attention for further investigations. This includes safety assessments of TP-PDT and UCNP-PDT, which contain toxicity tests of synthesized PS, dosage, and duration treatment within the human body.

## Figures and Tables

**Table 2:** TPE-PDT for the treatment of different cancers, either in in vitro or in vivo studies.

**In Vitro**					
	**Study**	**Photosensitizer (PS)**	**Usage of Light and** **Energy (J)**	**Consequence**	**Reference**
1	Evaluation of one- and two-photon activated photodynamic therapy with pyropheophorbide-a methyl ester in human cervical, lung, and ovarian cancer cells	Pyropheophorbide-a methyl ester (MPPa)	A laser at 800 nm through 2-γ excitation with 0.06 J/cm^2^.	Pyropheophorbide-a methyl ester was a potent photosensitizer for both 1- and 2-γ activated PDT with potential applications for difficult-to-treat tumors by conventional therapies.	[53]
2	Metal complexes for two-photon photodynamic therapy: A cyclometallated iridium complex induces two-photon photosensitization of cancer cells under near-IR light	Iridium complexes, [Ir(N^C)_2_(N^N)]^+^	A laser at 760 nm with 3.6 mW/cm^2^.	Iridium complexes displayed high PS activity killing cancer cells under NIR two-photon excitation (760 nm), which along with its photo-stability indicated potential future clinical application.	[54]
3	Sulfonated aluminum phthalocyanines for two-photon photodynamic cancer therapy: The effect of the excitation wavelength	Sulfonated aluminum phthalocyanine (AlPcS)	A laser at 750 nm with 75 mW/cm^2^.	AlPcS was a PS with good potential for two-photon PDT of human nasopharyngeal carcinoma cells.	[55]
4	Two-photon photodynamic therapy by water-soluble self-assembled conjugated porphyrins	Water-soluble porphyrin self-assemblies	A laser at 1270 nm with 3 mW/cm^2^.	The photocytotoxicities of water-soluble porphyrin self-assemblies for HeLa cancer cells were evaluated, which was an effective PDT agent.	[56]
5	Triphenylamines induce cell death upon 2-photon excitation	Triphenylamines (TPAs)	A laser at 760 to 860 nm with 15 to 144 mW/cm^2^.	TPAs were compatible with 2-photon excitation to simultaneously trigger cell death, which was the relationship between their cellular localization and the cell death mechanism for cancers.	[57]
**In Vivo**					
1	Cancer-targeted azo dye for two-photon photodynamic therapy in human colon tissue	Methyl 2-(3-(dimethylamino)phenoxy)acetate	A laser at 770 nm two-photon treatment with 300 mW/cm^2^.	Methyl 2-(3-(dimethylamino)phenoxy)acetate generated ROS efficiently in live colon cancer tissues with high spatial selectivity.	[58]
2	New two-photon activated photodynamic therapy sensitizers induce xenograft tumor regressions after near-IR laser treatment through the body of the host mouse	Porphyrin and two covalently attached bis(diphenylamino)distyrylbenzene (MPA79)	A laser at 820 to 1100 nm with 600 to 800 mW/cm^2^	PDT sensitizers were used at a depth of 2 cm to produce excellent xenograft regressions, and the tumor response was consistent with known responses to single-photon-activated PDT.	[59]
3	Iridium(III)-based infrared two-photon photosensitizers: systematic regulation of their photodynamic therapy efficacy	Cyclometalated iridium(III) complexes	A low-power laser at 808 nm with 100 mW/cm^2^.	This was extremely effective in treating large, profoundly located solid tumors, and understanding the structure-activity relationship of Ir(III)-based PS in PDT.	[60]
4	Real-time monitoring of colorectal cancer location and lymph node metastasis and photodynamic therapy using fucoidan-based therapeutic nanogel and near-infrared fluorescence diagnostic–therapy System	Fucoidan-based theranostic nanogel (CFN-gel)	A laser at 660 nm with 20 mW/cm^2^.	CFN-gel with a high accumulation efficiency in colorectal cancer cells and high fluorescence signals in near-infrared light for a long period, and only CFN-gel delayed the growth rate of colorectal cancer in terms of its size in PDT.	[61]
5	Self-assembled organic nanomedicine enables ultrastable photo-to-heat converting theranostics in the second near-infrared biowindow	Nano-boron difluoride formazanate (Nano-BFF)	Exposure to NIR laser at 1000–1700 nm with 1 W/cm^2^.	Nano-BFF was an efficient theranostic agent to achieve photoacoustic imaging-guided deep-tissue photonic hyperthermia in the NIR-II window, achieving dramatic inhibition toward orthotopic hepatocellular carcinoma.	[62]
6	Water-soluble polythiophene for two-photon excitation fluorescence imaging and photodynamic therapy of cancer	Polythiophene (PTo)	A laser at 720 nm with 275 mW/cm^2^.	PTo was demonstrated to be capable of simultaneous cell imaging and photodynamic therapy under either one-photon or two-photon excitation modes against A549 cells and 3T3 cells.	[63]
7	NIR-II light-activated two-photon squaric acid dye with Type I photodynamics for antitumor therapy	Squaric acid nanoparticles (SQNPs)	A laser at 730 to 840 nm with 100 mW/cm^2^.	SQNPs exhibited irreversible cytotoxicity against hypoxic tumor in NIR-II light-excited two-photon PDT, resulting in ablation of apparent solid tumor.	[64]
8	Rational design of organic probes for turn-on two-photon excited fluorescence imaging and photodynamic therapy	Acetal terminated distyrylbenzene derivative (Ace-DSB)	A laser at 1150 nm with 80 mW/cm^2^.	Ace-DSB enhanced two-photon laser confocal scanning microscopic imaging and two-photon excited photodynamic therapy (2PE-PDT) for MCF-7 cancer cells and melanoma tumors.	[65]
9	Rationally designed ruthenium complexes for 1- and 2-photon photodynamic therapy	Ruthenium(II) polypyridine complexes	A laser at 800 nm with 10 mW/cm^2^.	Ruthenium(II) polypyridine complexes were phototoxic in various 2D monolayer cells and 3D multicellular tumor spheroids, and were able to eradicate a multi-resistant tumor inside a mouse model.	[66]
10	Hyaluronic acid-modified metal–organic framework for two-photon imaging-guided photodynamic therapy in triple negative breast cancer	ZrTc nano MOF with hyaluronic acid (ZrTc@HA)	A laser at 780 nm with 80 mW/cm^2^.	ZrTc@HA exhibited exceptional antitumor ability for triple-negative breast cancer with minimal toxicity.	[67]

**Table 3:** UCNP-PDT for the treatment of different cancers, either in in vitro or in vivo studies.

**In Vitro**					
	**Study**	**Photosensitizer (PS)**	**Usage of Light and Energy (J)**	**Consequence**	**Reference**
1	Near-infrared light activated upconversion nanoparticles (UCNP) based photodynamic therapy of prostate cancers: An in vitro study	MC540/ZnPc-UCNP@Au	Irradiated to a PS at 540 to 660 nm and a NIR laser at 980 nm with 25 mW/cm^2^.	An efficient nano platform was established, MC540/ZnPc-UCNP@Au, for superficial and deep-seated PC-3 prostate cancer cells.	[74]
2	The use of upconversion nanoparticles in prostate cancer photodynamic therapy	Upconversion immune-nanohybrids (UINBs)	Irradiated to a PS at 520 to 540 nm and a NIR laser at 980 nm with 12.14 mW/cm^2^.	The UINB system specifically detected prostate cancer cells with stable and background-free luminescent signals for highly sensitive prostate cancer cell detection.	[75]
3	Near-infrared light-triggered photodynamic therapy and apoptosis using upconversion nanoparticles with dual photosensitizers	Chlorin e6 and Rose Bengal with Er-doped UCNPs	Irradiated to a laser at 808 nm with 2.5 mW/cm^2^.	The ROS generation in a dual photosensitizer system was significantly higher than that in a single photosensitizer system, and PDT induced immunogenic apoptosis for cells or tissues.	[76]
4	Controllable assembly of upconversion nanoparticles enhanced tumor cell penetration and killing efficiency	Chlorin e6 (Ce6) with Poly(styrene-*co*-maleic anhydride UCNPs	Irradiated to a PS at 400 to 675 nm and a NIR laser at 980 nm with 2.5 mW/cm^2^.	UCNPs clustered with different sizes could facilitate a clear and deep understanding of nanoparticle-based delivery platform systems for cancer cell killing.	[77]
5	Upconversion in photodynamic therapy: plumbing the depths	NaYF_4_ nanoparticles doped with Yb^3+^ and Er^3+^ or with Tm^3+^ and Er^3+^ with UCNPs	A laser at 980 nm with 50 mW/cm^2^.	Dye-sensitized UCNPs and UCNPs coupled to PS allowed NIR light energy to be transduced into ROS leading to cell killing and tumor regression.	[12]
6	Facile assembly of functional upconversion nanoparticles for targeted cancer imaging and photodynamic therapy	Rose Bengal with UCNPs	A laser at 980 nm with 2 mW/cm^2^.	Nanocomposites were shown to target cancer cells specifically to suppress cancer cell growth in vitro.	[78]
7	Photosensitizer functionalized luminescent upconverting nanoparticles for efficient photodynamic therapy of breast cancer cells	Rose Bengal with UCNPs	Irradiated to a PS at 541 to 652 nm and a NIR laser at 980 nm with 20 mW/cm^2^.	The RB-lysine-UCNPs were promising for NIR PDT and suitable for the treatment of deep-lying breast cancer cells.	[79]
8	Plasmon-enhanced photodynamic cancer therapy by upconversion nanoparticles conjugated with Au nanorods	upconversion nanoparticles (UCPs) conjugated gold nanorods (AuNRs) with Methylene blue (MB)	Irradiated to a PS at 808 nm and a NIR laser at 980 nm with 1 mW/cm^2^.	UCP@SiO_2_:MB-NRs-FA was evaluated to enhance ROS production through plasmonic field enhancement and thus achieve high PDT therapeutic efficacy.	[80]
9	NIR excitation of upconversion nanohybrids containing a surface grafted Bodipy induces oxygen-mediated cancer cell death	UCNPs capped with a polyethylene glycol (PEG) and a diiodo-substituted Bodipy (IBDP)	Irradiated to a PS at 515–565 nm, 590–740 nm, and a NIR laser at 975 nm with 239 mW/cm^2^.	UCNP-IBDP@PEG nanohybrid was taken up by the SH-SY5Y human neuroblastoma-derived cells showing cytotoxicity, and 50% cancer cell death was observed after NIR irradiation.	[81]
**In Vivo**					
1	Photosensitizing deep-seated cancer cells with photoprotein-conjugated upconversion nanoparticles	KillerRed; KR with a cancer cell-targeted lead peptide (LP) and UCNPs	Irradiated to a PS at 550 nm and a NIR laser at 980 nm with 1 mW/cm^2^.	NIR light irradiation exhibited significant PDT efficacy in cancer cells located beneath porcine skin tissues up to a depth of 10 mm, as well as in vivo tumor xenograft mouse models.	[82]
2	Mesoporous silica-coated upconversion nanoparticles assisted photodynamic therapy using 5-aminolevulinic acid: mechanistic and in vivo studies	UCNPs with 5-aminolevulinic acid (UCNPs-5-ALA)	A laser at 980 nm with 500 mW/cm^2^.	Mice treated with UCNPs-5-ALA did not possess any in vivo cytotoxicity and were irradiated to reduce 75% of the tumor size.	[83]
3	PDT-active upconversion nanoheaters for targeted imaging guided combinatorial cancer phototherapies with low-power single NIR excitation	UCNP loaded with Rose Bengal	Irradiated to a PS at 561 nm and a NIR laser at 975 nm with 400 mW/cm^2^.	The highly stable UCNP@Tf-RB exhibited excellent ROS/heat generating capability demonstrated by DPBF degradation and photothermal imaging for cancers, respectively.	[84]
